# First report of monepantel *Haemonchus contortus* resistance on sheep farms in Uruguay

**DOI:** 10.1186/s13071-014-0598-z

**Published:** 2014-12-17

**Authors:** América E Mederos, Zully Ramos, Georgget E Banchero

**Affiliations:** Beef and Wool Program, National Research Institute for Agriculture (INIA), Ruta 5 Km 386, Tacuarembó, 45000 Uruguay; Beef and Wool Program, National Research Institute for Agriculture (INIA), La Estanzuela, Ruta 50 Km 11, Colonia, Uruguay

**Keywords:** Sheep, Monepantel, Anthelmintic resistance, *Haemonchus contortus*, Uruguay

## Abstract

**Background:**

On two farms it was noted that after routine treatment with monepantel, fecal egg counts failed to drop. This was accompanied by lambs mortality due to *Haemonchus contortus* infection. The aim of this work was to evaluate the efficacy of monepantel to control gastrointestinal nematodes (GIN) in two sheep farms, in Uruguay.

**Findings:**

A Fecal Egg Count Reduction Test (FECRT) was subsequently performed at the Experimental Stations Glencoe of INIA Tacuarembó (Farm 1) and Sheep Unit of INIA La Estanzuela (Farm 2) using the World Association for the Advancement of Veterinary Parasitology guidelines. On Farm 1 the FECRT was performed using 6–8 month old Corriedale or Merino Dohne x Corriedale male lambs naturally infected with GIN. On day 0 pre-treatment, three groups of 15 lambs each were selected, blocked by fecal egg count level (FEC) and randomly assigned to one of the following: Group 0 = untreated control, Group 1 = treated with monepantel (Zolvix®, Novartis Animal Health Inc.) from stock previously purchased; Group 2 = treated with monepantel from stock provided by the supplier, at the recommended dose of 2.5 mg/kg of body weight. Fecal samples were collected directly from the rectum from each lamb on day 0 and on day 9 post-treatment. On Farm 2, the FECRT was conducted on a group of 8 month old male lambs Milchschaff x Finn. At this farm, 10 lambs were randomly allocated to be treated with monepantel (Group 1) and 10 lambs were randomly allocated to remain as untreated control (Group 0) using the same protocols as Farm 1.

On farm 1 the FECR was 0.0% (95% CI = 0.0 – 49.0) and 42.0% (95% CI = 0.0 – 75.0) for Group 1 and Group 2 respectively. For Farm 2, the FECR was 82.1% (95% CI = 36.0 – 99.0). *Haemonchus* spp was the resistant genus.

**Conclusions:**

Poor effcicacy of monepantel in treating GIN parasites was demonstrated on both farms.

## Findings

### Background

In Uruguay, ovine production plays a very important role in the economy. Parasitism due to gastrointestinal nematodes (GIN) is one of the most important health constraints affecting sheep rearing operations and its control has relied primarily on the use of chemical drugs. As a result, anthelmintic resistance (AR) is a wide-spread phenomenon amongst sheep farms in this country. A national survey conducted between 1994 and 1995 to quantify the prevalence of anthelmintic resistance (AR) in sheep GIN [1], revealed that benzimidazole (BZ), levamisole (LEV) and ivermectin (IVM) resistance was present on 80%, 71% and 1.2% respectively, of the studied sheep farms (n = 252). Subsequently, several reports from different diagnostic laboratories established that the prevalence of AR continues to escalate. In 2005, results from a sample of 130 sheep farms revealed that 89% had resistance to IVM, 82% to LEV, 89% to closantel and 29% to moxidectin [2]. In both studies, *Haemonchus* sp and *Trichostrongylus* spp were the main genera reported as resistant.

After many years, a new class of anthelmintic, the amino-acetonitrile derivative monepantel (Zolvix, Novartis Animal Health Inc.) was developed for the control of GIN in sheep [3]. Monepantel was first available in New Zealand in 2009 and became commercially available in Uruguay in 2010. The first report of AR to monepantel was reported from New Zealand in 2013 as a lack of efficacy against *Teladorsagia circumcincta* and *Trichostrongylus colubriformis* in goats [4].

The objective of this study was to evaluate the suspicion of poor efficacy of monepantel against gastrointestinal nematodes on two sheep farms at the National Research Institute for Agriculture (INIA), in Uruguay.

## Methods

### The studied farms

The study was conducted at the Experimental Stations Glencoe of INIA Tacuarembó (Farm 1), and INIA La Estanzuela (Farm 2), Colonia, Uruguay. Farm 1 is an extensive sheep and cattle production farm, occupying 1300 ha and located southwest of Uruguay (32° 00’24”S, 57° 08’ 01” W). The farm maintains three sheep flocks: Australian Merino (n = 977), Corriedale (n = 258) and Merino Dohne and their crosses (n = 883). The gastrointestinal parasite control program is based on fecal egg count monitoring and anthelmintic treatment when indicated by high fecal egg counts. History of monepantel use on this farm was investigated to describe possible risk factors present for development of AR.

Farm 2 is located in the southwest region of Uruguay (34°19´57´´S, 57°40´07´´W) and is an intensive sheep production system (n = 2250 to 2500) occupying 100 ha with one flock comprised of multiple breeds mainly Milchschaf, Finn, Texel and Polwarth. Gastrointestinal parasite control is based on FAMACHA scoring [5,6] and targeted selective treatment of sheep scoring “4” or “5”. History of monepantel use on this farm was also investigated.

Multidrug AR (benzimidazole, levamisole, ivermectin and closantel) was diagnosed on both farms by FECRT performed on a routine base, with *Haemonchus contortus* being the main gastrointestinal nematode diagnosed (unpublished data).

Sheep trade between both farms has been rare.

### Fecal egg count reduction test

Fecal egg count reduction tests (FECRT) were carried out using the World Association for the Advancement of Veterinary Parasitology (WAAVP) guidelines [7,8]. On Farm 1 at the initial visit designated day −6 (May 14^th^, 2014), a total of 123 Corriedale and Merino Dohne × Corriedale 6–8 month old castrated male lambs naturally infected with gastrointestinal nematodes and not having been treated with any anthelmintic in 30 days, were weighed and individual fecal samples were taken directly from the rectum. The samples were identified with ear tag number and transported refrigerated to the laboratory and processed immediately after arrival. Results were used to assign lambs to treatment groups.

On day 0 pre-treatment (May 20^th^, 2014), three groups of 15 lambs each were selected, blocked by fecal egg count level (FEC) and randomly assigned to one of the following: Group 0 = untreated control (G0), Group 1 = treated with monepantel (Zolvix®, Novartis Animal Health Inc.) from stock previously purchased (G1); Group 2 = treated with monepantel (Zolvix®, Novartis Animal Health Inc.), from stock provided by the supplier (G2).

Lambs assigned to G1 and G2 were drenched orally using a syringe at the corresponding drug dose according to their body weight, while lambs from G0 received no treatment. At the same time, fecal samples were collected directly from the rectum from each lamb, placed in a plastic bag, sealed after air removal, identified with ear tag, placed in a cooler and transported to the lab.

On day 9 post-treatment (May 29^th^, 2014) a second fecal sample from all lambs was taken and transported following the same procedure previously described.

At Farm 2, the FECRT was conducted to evaluate five drugs, but here we will describe only the monepantel evaluation. For this purpose, eight month old male lambs Milchschaff × Finn were randomly selected (n = 20) and 10 were randomly allocated to remain untreated as a control (Group = 0) and 10 lambs were randomly allocated to be treated with monepantel (Group = 1). From here, everything was performed according to the protocol described above.

All samples were processed at INIA Tacuarembó Animal Health Laboratory. Fecal egg counts were performed using a modified McMaster technique [9] with a lower detection limit of 50 eggs per gram. Coprocultures were performed using pooled fecal samples: the pre-treatment sampling at day 0 (G0, G1 and G2 combined), and subsequently one for each group at the post-treatment sampling at day 9. The cultures were placed at 27°C during 10 days and then transferred to Baermann apparatus, recovered after 24h and kept refrigerated at 4°C until analysis using standard identification keys [9,10].

### Analysis and results interpretation

The formula described by Dash (1988) [11] was used to estimate flock FECR, based on arithmetic means fecal egg counts in controls (C) and treated (T) animals:

$$ \mathrm{FECR}=100\times \left(1\hbox{--} \left[\mathrm{T}2/\mathrm{T}1\right]\left[\mathrm{C}1/\mathrm{C}2\right]\right), $$

and 95% Confidence Intervals were estimated using the BootStreat program [12].

## Results

### Farm 1

At day −6 pre-treatment the mean FEC after sorting the groups were 3010, 3530 and 3717 eggs per gram and the mean body weight 29 kg, 27 kg and 28 kg for the lambs of G0, G1 and G2, respectively.

The results presented in Table 1, supports the hypothesis that monopantel lacks sufficient efficacy to reduce the FEC burden in the studied farms and that anthelmintic resistance to monepantel exists in that parasite population.

**Table 1:** **Results of the mean fecal egg counts pre and post-treatment for all groups and %FECR for the evaluated anthelmintic drugs and the 95% confidence intervals (CI)**

	**FEC Pre-treatment**	**FEC Post-treatment**	**%FECR (95% CI)**
**Farm 1**			
G0 = Control	3307	5490	NA
G1 = Monepantel^1^ Lab	3020	5132	0.0 (0.0 – 49.0)
G2 = Monepantel New	5117	5114	42.0 (0.0 – 75.0)
**Farm 2**			
G0 = Control	6875	6345	NA
G1 = Monepantel Lab	2880	475	82.1 (36.0 – 99.0)

FEC = fecal egg counts; %FECR = percentage fecal egg counts reduction; NA = not applicable.

^1^Zolvix (Novartis Animal Health Inc.).

### Farm 2

The results presented in Table 1, demonstrated that both the %FECR and the 95% confidence interval were below the limit established for adequate efficacy by the WAAVP guidelines. Results from coprocultures revealed that *Haemonchus* spp. was the main genus that developed in monepantel groups (see Table 2).

**Table 2:** **Results of the percentage of gastrointestinal nematodes genera identified from the coprocultures at both farms**

**Group**	**Time**	***Haemonchus*** **sp. %**	***Trichostrongylus*** **sp. %**	***Oesophagostumum*** **sp. %**
***Farm 1***				
Control	Day 0	86	11	3
Control	Day10	92	7	1
Monepantel^1^ Lab	Day10	100	0	0
Monepantel New	Day10	100	0	0
***Farm 2***				
Control	Day 0	4	85	11
Control	Day10	28	52	20
Monepantel Lab	Day10	93	3	4

^1^Zolvix (Novartis Animal Health Inc.).

### History of Monepantel use

Historical use of monepantel was considered retrospectively as a possible risk factor for development of anthelmintic resistance. On Farm 1, the first treatment with monepantel was given in March 2011 (autumn); a second treatment was given in August and a third in December to the whole ewe flocks. Subsequently, during 2012, a single drench was administered to all the lambs and all breeding ewes received two drenches (before lambing and nursing). However, during 2013 the drug was used more frequently, being all lambs and all ewes the group that received three treatments based on fecal egg counts.

On Farm 2, monepantel use started in May 2011, but here sheep and lambs were selectively treated based on routine FAMACHA scoring for anemia and the proportion of the flock that required treatment varied from 5% to 10%. Paddocks management in both farms is very complex in comparison with commercial sheep farms, due to the various production and experimental trials performed. Therefore, retrospective unbiased results about potential causal factors could not be obtained.

## Discussion

The results presented above demonstrated that on two farms, a multidrug resistant strain of *Haemonchus contortus* (unpublished observations) developed apparent resistance to monepantel as evidence by treatment failure assessed using a FECRT. Resistance to monepantel has also been reported in New Zealand [4,5], but in that case monepantel failed to control *Teladorsagia circumcincta* and *Trichostrongylus colubriformis* in goats. In New Zealand monepantel was licensed in 2009 and resistance was reported four years later, similar to what has now happened in Uruguay since monepantel was licensed in 2010 and resistance detected in 2014. Previous research has demonstrated an AAD mutant gene present in a sub-population of *H. contortus* [3,13]. The results of this investigation support the presence of this gene in the field through its apparent phenotypic expression on these two farms. To confirm the presence of this gene, *Haemonchus* spp. isolates from the coprocultures and adult worms would need to be further examined. In addition to the FECRT, other methods to detect multidrug anthelmintic resistence might be explored, as recently proposed by Roeber et al. [14].

On both Farm 1 and Farm 2, the frequency of monepantel use was low prior to experiencing apparent treatment failure. High frequency of treatments and lack of population in refugia are the main risk factors for AR development most commonly incriminated in the literature. Based on the history available, these factors do not seem to be important here. As mentioned above, on Farm 1, monepantel was seldom used from 2011 to 2012 and applied three times to all animals from the same flock during 2013. On Farm 2, all treatments were applied using targeted selective treatment based on FAMACHA scoring, thus not eliminating refugia due to massive treatment. This is in concordance with a recent study that highlighted the lack of unbiased scientific evidence for risk factors associated with the development of AR in sheep gastrointestinal nematodes [15].

## Conclusions

The present situation of anthelmintic resistance in Uruguay is becoming aggravated with the early development of resistance to monepantel by *Haemonchus* spp. (putative *Haemonchus contortus*). Further molecular studies are necessary to understand the mechanism of monepantel resistance, allowing early detection to develop strategies to prevent the spread of resistant worms.
